# Targeting interleukin-6 trans-signaling to improve post-stroke inflammation: A precision immunotherapy perspective

**DOI:** 10.4103/NRR.NRR-D-25-01158

**Published:** 2026-01-27

**Authors:** Kate Mendoza, Dustin T. Nguyen, Anjali Chauhan

**Affiliations:** Department of Neurology, University of Texas McGovern Medical School at Houston, Houston, TX, USA

**Recognizing stroke recovery through an immunological lens:** Stroke has traditionally been viewed as an acute neurological event resulting from vascular occlusion and subsequent neuronal death. However, mounting evidence now supports a broader perspective: stroke triggers a dynamic neuroinflammatory response that begins within hours of injury and can persist through the subacute and chronic phases. These sustained immune responses play a critical role in shaping long-term recovery.

One cytokine that plays a role in both injury and repair is interleukin-6 (IL-6). After stroke, IL-6 is secreted by multiple cell types, including microglia, astrocytes, macrophages, endothelial cells, and T cells. IL-6 signals through two distinct modes: classical signaling, via membrane-bound IL-6 receptor (mIL-6R) and gp130, which is generally associated with reparative, anti-inflammatory effects; and trans-signaling, in which IL-6 binds soluble IL-6R (sIL-6R) to activate gp130 on a broad range of cells, often driving pro-inflammatory responses. IL-6 trans-signaling has been linked to worse outcomes in experimental stroke and traumatic brain injury (Hall et al., 2025). These mechanistic distinctions offer a therapeutic opportunity: selectively inhibiting IL-6 trans-signaling while preserving classical signaling may suppress harmful inflammation without impairing recovery-related processes, a strategy that is especially promising in stroke, where timing and cellular context critically determine outcomes.

**Interleukin-6 signaling — a dual-edged sword in neuroinflammation:** The dual role of IL-6 in stroke pathophysiology presents both a challenge and an opportunity. On one hand, IL-6 is a potent driver of systemic and neuroinflammation, contributing to secondary tissue damage and poorer outcomes in stroke patients. On the other hand, it may support neural repair through localized effects within the brain (Pawluk et al., 2025). A preclinical study has shown that IL-6 can promote angiogenesis and neurogenesis; intracerebroventricular administration of IL-6 has demonstrated neuroprotective effects in rodent models of stroke (Pawluk et al., 2025).

Clinically, however, elevated circulating IL-6 levels are consistently associated with worse stroke outcomes. A meta-analysis of 11 studies reported a 19% increased risk of ischemic stroke for each standard deviation increase in IL-6 (Papadopoulos et al., 2022). Similarly, a prospective cohort study has linked high IL-6 levels to increased risk of recurrent vascular events, vascular mortality, and all-cause mortality (Whiteley et al., 2011). A large-scale genetic study from the MEGASTROKE and CARDIoGRAMplusC4D consortia has further shown that genetically downregulated IL-6 signaling is associated with a lower risk of ischemic stroke, including both large artery and small vessel subtypes (Georgakis et al., 2020).

These findings have prompted interest in IL-6 as a therapeutic target. Tocilizumab, a monoclonal antibody that blocks both classical and trans-signaling via IL-6 receptor inhibition, has shown cardioprotective effects in clinical trials by dampening inflammation (Huse et al., 2022). However, its broad mechanism of action may inadvertently suppress beneficial IL-6 signaling in the brain and has been associated with increased infection risk, an important concern in stroke patients, who often exhibit post-stroke immunosuppression. Together, these limitations highlight the need for more selective strategies. Inhibiting IL-6 trans-signaling specifically while preserving classical signaling could achieve targeted immunomodulation that reduces harmful inflammation without impairing endogenous repair mechanisms. Agents such as soluble (s) gp130Fc offer a promising path forward in this regard, providing a more precise and potentially safer approach to improving stroke outcomes.

**Figure 1:**
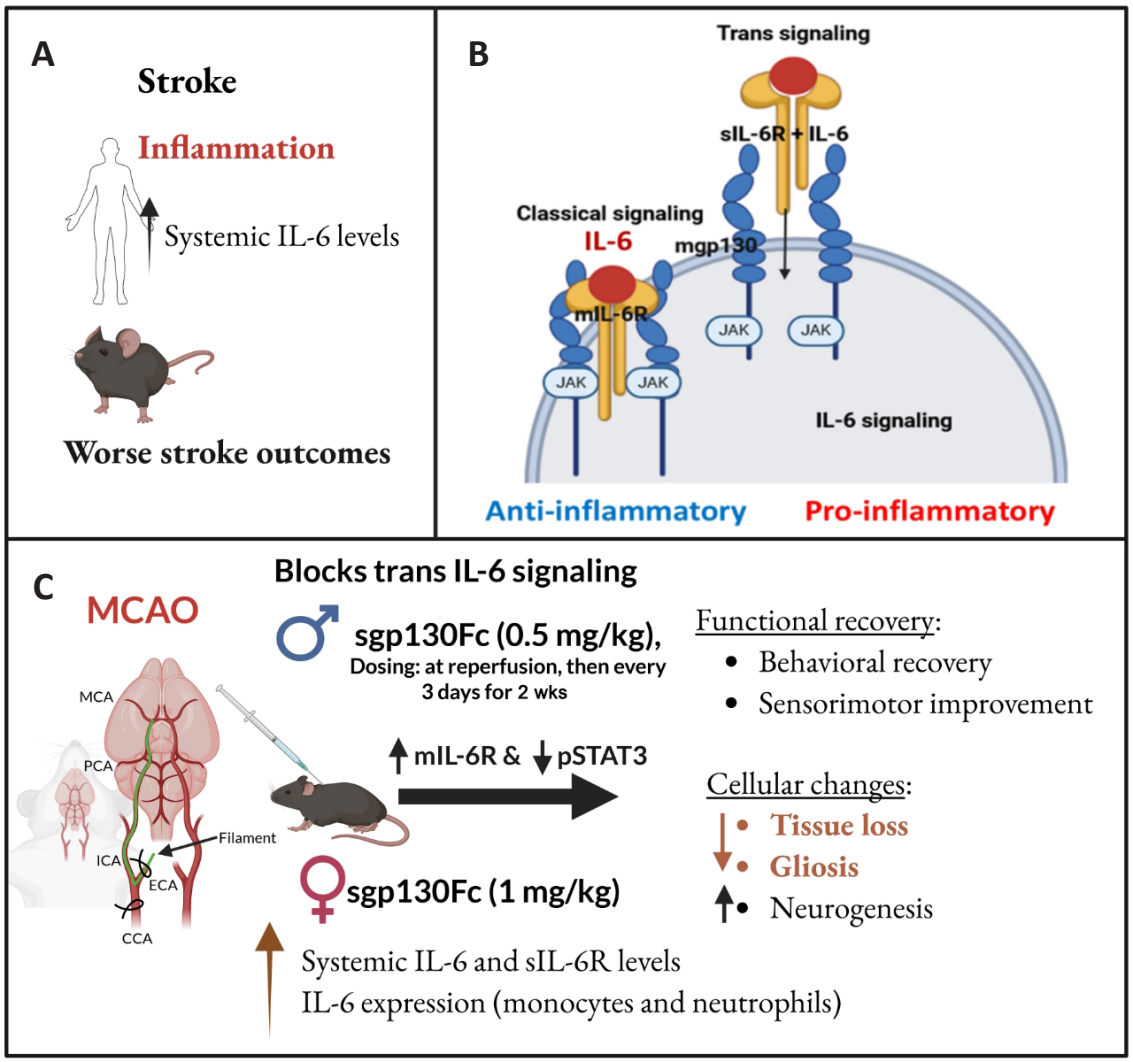
Inhibition of IL-6 trans-signaling improves stroke outcomes in a sex- and dose-dependent manner. (A) Stroke leads to an increase in systemic IL-6 levels, which are associated with worse outcomes. (B) Classical and trans IL-6 signaling. (C) Selective inhibition of IL-6 trans-signaling using sgp130Fc reduces neuroinflammation and promotes functional recovery after stroke. Created in BioRender. Chauhan, A. (2025) https://BioRender.com/cx5oamf. CCA: Common carotid artery; ECA: external carotid artery; ICA: internal carotid artery; IL: interleukin; MCA: middle cerebral artery; MCAO: middle cerebral artery occlusion; mIL-6R: membrane-bound interleukin-6 receptor; PCA: posterior cerebral artery; pSTAT3: phosphorylated STAT3; sIL-6R: soluble interleukin-6 receptor.

**Study highlights — soluble (s) gp130Fc as a selective modulator of interleukin-6 trans-signaling:** Sgp130Fc is a biologic that selectively inhibits IL-6 trans-signaling while preserving classical signaling. Its efficacy in reducing inflammation and promoting tissue repair has been demonstrated across cardiovascular and neuroinflammatory models (Schuett et al., 2012; Burton et al., 2013; Li et al., 2023). In the brain, sgp130Fc attenuates sickness behavior and modulates neuroimmune responses, making it a compelling candidate for stroke (Burton et al., 2013). In our recent study, we evaluated sgp130Fc for the first time in experimental stroke and showed sex- and dose-dependent benefits (Hall et al., 2025). Treatment improved long-term behavioral outcomes, including cognitive and sensorimotor function, and was associated with reduced neuroinflammation and enhanced neurogenesis (**Figure 1**). Notably, these effects occurred without increasing infection risk, as systemic markers such as IL-6 and lipopolysaccharide-binding protein remained stable.

An unanticipated finding was a dose-dependent difference between sexes. Whereas low-dose sgp130Fc was sufficient to improve recovery in males, females required a higher dose to achieve comparable effects. Subsequent analyses revealed higher post-stroke systemic IL-6 and sIL-6R levels in females, suggestive of greater IL-6 trans-signaling. Although these observations were not the primary focus of the study, they emphasize how sex can shape treatment responsiveness and may help refine dosing in future work. By preserving the reparative signaling of IL-6 while dampening its pro-inflammatory effects, sgp130Fc offers a more refined approach to post-stroke immune modulation. These findings reinforce that IL-6 trans-signaling is a modifiable determinant of stroke outcome and support further exploration of pathway-specific immunotherapies in translational models.

**Mechanistic insight into post-stroke recovery — lessons from sgp130Fc:** Selective inhibition of IL-6 trans-signaling with sgp130Fc at 7 days after middle cerebral artery occlusion produced a coherent immune phenotype across the periphery and brain that aligns with functional benefits. In the spleen, sgp130Fc preserved mIL-6R on F4/80⁺ macrophages and Ly6Chi monocytes in both sexes; neutrophils were unchanged. A sex-divergent T-cell effect emerged: males showed higher mIL-6R on CD3^+^ T cells, whereas females did not. These data suggest sgp130Fc limits post-stroke IL-6R shedding on select peripheral myeloid and (in males) T-cell compartments, thereby restricting formation of IL-6/sIL-6R complexes and biasing signaling away from trans- and back toward classic signaling pathways.

In the brain, sgp130Fc increased the proportion of infiltrating macrophages (CD45^hi^CD11b^+^F4/80^+^) that retain mIL-6R in both sexes, while reducing phosphorylated STAT3 within these cells. Given prior reports that sgp130Fc dampens STAT3 activation across inflammatory conditions, the observed reduction in phosphorylated STAT3 in brain macrophages is consistent with on-target suppression of trans-signaling. Cerebrovascular injury models suggest that IL-6R^+^, repair-associated Iba1^+^ cells may support vascular repair and recovery, potentially via IL-6 supplied by inflammatory monocytes; depleting these monocytes can impair the reparative response (Choi et al., 2023). By **~**day 7 post-injury, infiltrating monocytes tend to acquire microglia-like features. Our findings fit a parsimonious model: by sequestering IL-6/sIL-6R complexes, sgp130Fc preserves mIL-6R on infiltrating macrophages and key peripheral subsets, restrains maladaptive STAT3 activation, and maintains a pool of IL-6R^+^ repair-competent macrophage-like cells in the injured brain. This cell-type re-balancing of IL-6 signaling may help explain, in part, the long-term improvements observed, while acknowledging that additional mechanisms likely contribute.

**Beyond lesions — the emerging paradigm of functional recovery and plasticity:** Infarct volume has long been a standard endpoint in preclinical stroke research. Although lesion size remains an important marker of acute injury, it fails to fully reflect the complexity of recovery, especially in models such as young mice that exhibit robust plasticity. In this article, sgp130Fc significantly reduced infarct volume when administered at reperfusion. However, improvements in cognitive performance, neurogenesis, and neuroinflammation suggest that its benefits extend beyond simple lesion containment.

Sgp130Fc-treated animals displayed marked reductions (*P* < 0.05) in peri-infarct gliosis, with fewer Iba1^+^ microglia and GFAP^+^ astrocytes compared to vehicle-treated stroke mice, indicating that inhibition of peripheral IL-6 trans-signaling dampens neuroinflammation. These histological changes were mirrored by enhanced functional outcomes; sgp130Fc-treated mice performed better (*P* < 0.05) on hippocampal-dependent tasks such as the Barnes maze and showed improved sensorimotor coordination and nest-building performance than vehicle-treated stroke mice. Interestingly, sgp130Fc treatment did not alter angiogenesis; instead, the most pronounced effects were observed in the subventricular zone, where neurogenesis markers were significantly elevated. The sgp130Fc-treated group exhibited higher numbers (*P* < 0.05) of doublecortin, DCX^+^ immature neurons and a trend toward increased 5-bromo-2′-deoxyuridine, BrdU^+^ proliferating cells, along with greater subventricular zone IL-6 mean fluorescence intensity relative to vehicle controls. These findings suggest that selective blockade of peripheral IL-6 trans-signaling preserves or even enhances central IL-6 activity, thereby promoting classical IL-6/mIL-6R signaling in neurogenic niches.

Flow cytometric analyses further revealed increased mIL-6R expression on live brain cells in sgp130Fc-treated mice, with no change in membrane gp130 expression, indicating intact central IL-6 signaling. In contrast, the spleen showed only a modest, nonsignificant rise in mIL-6R, consistent with partial inhibition of peripheral receptor shedding. Collectively, these results suggest that sgp130Fc exerts dual actions: reducing peripheral inflammatory signaling while preserving central IL-6/mIL-6R pathways that support neurogenesis and plasticity. Together, these data illustrate a broader shift in how stroke therapies should be evaluated. By moving beyond lesion volume to encompass behavioral recovery, neuroinflammation, and neural regeneration, sgp130Fc exemplifies a class of interventions that do not merely protect the brain from injury but actively engage its endogenous repair mechanisms.

**Translational outlook — precision immunotherapy for stroke recovery:** The limited success of anti-inflammatory therapies in stroke stems in part from a lack of specificity both in pathway targeting and patient selection. Broad immunosuppression risks undermining repair processes or worsening infection risk. Sgp130Fc provides a blueprint for precision immunotherapy: a selective, pathway-specific strategy that targets a validated mechanism of injury while preserving the capacity for neural recovery. Our findings illustrate how biological variability, including sex-related differences in IL-6/sIL-6R levels, can shape treatment response. Even when not initially anticipated, they reinforce a precision framework: tailor anti-inflammatory therapy to an individual’s signaling milieu rather than applying uniform blockade. By inhibiting IL-6 trans-signaling alone, sgp130Fc avoids the pitfalls of global IL-6 blockade. In this study, it did not increase infection risk markers (e.g., lipopolysaccharide-binding protein), and its efficacy varied predictably with sex and dose, highlighting the importance of personalized therapeutic thresholds.

**Future directions, unanswered questions and testable hypotheses:** While sgp130Fc demonstrates the feasibility of selectively modulating IL-6 trans-signaling to promote recovery, key mechanistic and translational questions remain.

• Does preserving central mIL-6R signaling drive repair? Hypothesis: sgp130Fc increases brain mIL-6R^+^ infiltrating macrophages, reduces IL-6 trans signaling, and thereby enhances subventricular zone neurogenesis and synaptic remodeling.

• Biomarker prediction of response: Hypothesis: Benefit from sgp130Fc correlates with early (24–72 hours) IL-6:sIL-6R ratios and related markers (e.g., sgp130, C-reactive protein, fibrinogen) in plasma/cerebrospinal fluid, measured longitudinally with behavioral outcomes.

• How do age, comorbidity, and sex shape dosing? Hypotheses: (a) Aging/comorbidity shifts the effective dose window via immune senescence and systemic inflammation; (b) Sex hormones and sex-chromosome context modulate sIL-6R shedding and mIL-6R stability, yielding sex-specific biomarker profiles and necessitating age- and sex-aware dosing.

• Is a delayed start still effective? Hypothesis: Delayed-start regimens (initiated hours post-reperfusion) retain efficacy, expanding the therapeutic window and translational relevance.

Together, testing these hypotheses will convert sgp130Fc from proof-of-concept into a biomarker-guided, personalized immunotherapy for post-stroke recovery, centered on selective IL-6 trans-signaling inhibition.

**Limitations and alternative mechanisms:** Our outlook is constrained using young, otherwise healthy animals and a dosing paradigm that begins at reperfusion; both factors may overestimate effect size relative to aged or comorbid populations and do not establish a delayed therapeutic window. Although we interpret the data through the lens of IL-6 trans-signaling, parallel and interacting pathways likely contribute to recovery. Receptor biology also introduces uncertainties: ADAM10/17-dependent shedding and local sIL-6R availability could affect mIL-6R classic signaling. In addition, we did not establish the longitudinal stability of IL-6/sIL-6R in plasma/cerebrospinal fluid, which will require paired sampling across defined windows. Finally, while infection-associated markers (e.g., lipopolysaccharide binding protein) did not increase in our study, context-specific immunosuppression remains a potential risk; future work will incorporate targeted infection investigations to directly evaluate risk. Addressing these limitations, particularly in aged/comorbid models and with biomarker-guided, delayed-start designs, will be essential to determine whether selective IL-6 trans-signaling modulation is necessary and sufficient for functional recovery in clinically relevant settings.

**Conclusion:** Selective inhibition of IL-6 trans-signaling emerges as a promising strategy to improve post-stroke recovery by attenuating damaging inflammation while preserving reparative classical signaling. In experimental stroke, sgp130Fc improved cognitive and sensorimotor outcomes, reduced neuroinflammation, and enhanced neurogenesis without increases in infection markers, consistent with a pathway-selective mechanism. Variability in benefit shaped by dose and sex-related differences in IL-6/sIL-6R supports a precision approach guided by biomarkers rather than uniform blockade. Next steps include validation in aged and comorbid models and longitudinal profiling of IL-6 signaling to refine dosing windows and identify likely responders, advancing pathway-specific immunotherapy toward clinical translation.

*This work was funded by the National Institute of Neurological Disorders and Stroke (1R01NS128412-01A1) to AC.*

*Presentation at a meeting: ISC 2025, organized by International Stroke Conference, at Los Angeles, USA, on February 2025.*
